# Genome-Wide Identification and Characterization of the bHLH Transcription Factor Family in Pepper (*Capsicum annuum* L.)

**DOI:** 10.3389/fgene.2020.570156

**Published:** 2020-09-25

**Authors:** Zhishuo Zhang, Juan Chen, Chengliang Liang, Feng Liu, Xilin Hou, Xuexiao Zou

**Affiliations:** ^1^State Key Laboratory of Crop Genetics and Germplasm Enhancement, Key Laboratory of Biology and Germplasm Enhancement of Horticultural Crops in East China, Ministry of Agriculture, Nanjing Agricultural University, Nanjing, China; ^2^Hunan Vegetable Research Institute, Changsha, China; ^3^College of Horticulture, Hunan Agricultural University, Changsha, China

**Keywords:** genome-wide, pepper, bHLH transcription factor family, phylogenetic relationships, expression analysis

## Abstract

Plant basic helix–loop–helix (bHLH) transcription factors are involved in the regulation of various biological processes in plant growth, development, and stress response. However, members of this important transcription factor family have not been systematically identified and analyzed in pepper (*Capsicum annuum* L.). In this study, we identified 122 *CabHLH* genes in the pepper genome and renamed them based on their chromosomal locations. *CabHLHs* were divided into 21 subfamilies according to their phylogenetic relationships, and genes from the same subfamily had similar motif compositions and gene structures. Sixteen pairs of tandem and segmental duplicated genes were detected in the CabHLH family. *Cis*-elements identification and expression analysis of the *CabHLHs* revealed that they may be involved in plant development and stress responses. This study is the first comprehensive analysis of the *CabHLH* genes and will serve as a reference for further characterization of their molecular functions.

## Introduction

The bHLH transcription factor (TF) family, named for its basic helix–loop–helix (bHLH) structure, is the second largest class of TFs and is widely distributed in animals, plants, and microorganisms (Guo et al., 2008). The bHLH domain consists of approximately 60 amino acids and is divided into a basic amino acid region and a helix–loop–helix region (Toledo-Ortiz et al., 2003). The basic region is located on the *N*-terminal side of the bHLH domain and is approximately 15 amino acids in length. These amino acids are mainly responsible for binding to *cis*-elements in DNA. The HLH region is located on the *C*-terminal side of the domain, consists of approximately 40 amino acids, and promotes the formation of homo- and heterodimer complexes (Murre et al., 1989; Ferre-D’Amare et al., 1994).

According to their evolutionary relationships, DNA binding abilities, and functional characteristics, bHLH proteins in animals have been divided into six groups, A–F (Atchley and Fitch, 1997). Many of the plant bHLH proteins that have been identified belong to Group B (Sailsbery and Dean, 2012). According to classification criteria developed in animals, the 133 *bHLH* genes found in *Arabidopsis thaliana* have been divided into 12 subfamilies based on conserved amino acids at specific positions and on the presence or absence of additional conserved domains (Heim et al., 2003). Fourteen new bHLH TFs were subsequently discovered and further divided among 21 subfamilies, but this classification was limited to higher terrestrial plants (Toledo-Ortiz et al., 2003). As more family members were identified in species such as moss and seaweed, bHLH TFs were subdivided into 32 subfamilies (Carretero-Paulet et al., 2010). At present, the classification of the plant bHLH TF family is not clearly defined, and there are no corresponding names for each subfamily across species.

In plants, bHLH TFs are involved in various signal transduction and anabolic pathways, such as light signal transduction (Duek and Fankhauser, 2003; Castillon et al., 2007), anthocyanin synthesis (Ludwig et al., 1989; Goodrich et al., 1992; Quattrocchio et al., 1993; Nesi et al., 2000; Sakamoto et al., 2001), tryptophan synthesis (Smolen et al., 2002) and gibberellin synthesis (Arnaud et al., 2010). They also modulate stress responses, including responses to low temperature (LTR) (Feng et al., 2012; Lin et al., 2014; Xu et al., 2014), heat (Ko et al., 2009), drought (Li et al., 2007; Seo et al., 2011), and salt (Murre et al., 1989; Zhou et al., 2009; Liu et al., 2014). For example, *Arabidopsis* TT8 (*AtbHLH42*) typically regulates the synthesis of anthocyanins and procyanidins of vegetative organs through the formation of MYB-bHLH-WD (MBW) complexes, specifically the TT2-TT8-TTG1 complex (Baudry et al., 2004). *AtNIG1* was the first bHLH TF shown to be involved in the plant salt stress signaling pathway, and *Arabidopsis* atnig1-1 knockout mutants are significantly more sensitive to salt stress than wild-type plants (Kim and Kim, 2006). *AtbHLH122* overexpressing plants have stronger salt and anti-osmotic capacity than wild-type plants (Liu et al., 2014). The expression level of *AtbHLH92* is upregulated under NaCl, drought and cold stress (Jiang et al., 2009). In rice, *OsbHLH148* regulates the expression of genes associated with jasmonic acid signaling and serves as an initial response factor during drought stress, thereby participating in both drought and trauma responses (Kiribuchi et al., 2004). *OsbHLH1* can be specifically induced by cold stress, but is not induced by salt, PEG and ABA (Wang et al., 2003). *OsHLH006* participates in drought and wounding responses through the jasmonic acid signaling pathway (Kiribuchi et al., 2005).

Pepper (*Capsicum annuum* L.) is an economically important vegetable and the most widely grown cooking ingredient in the world. With the completion of the pepper genome sequence (Qin et al., 2014), genome-wide identification and classification of gene families can be performed to study genes that are critical for pepper growth and development. To date, a number of TF families have been characterized in pepper, such as the Dof (Wu et al., 2016) and Hsp70 (Guo et al., 2016) families. However, the pepper bHLH family has not been characterized previously. Here, we use a bioinformatics approach to identify and characterize members of the bHLH family in pepper. We report basic information about each gene, including its conserved domains, evolutionary relationships, chromosomal location, expression in various pepper tissues, and response to abiotic stress. These data provide a reference for further exploration of the molecular functions of *bHLH* genes in regulating pepper growth and stress responses.

## Materials and Methods

### Identification of the bHLH Gene Family

Annotated sequences of pepper and tomato genes were downloaded from the Solanaceae Genomics Network^1^, and annotated sequences of *Arabidopsis bHLHs* were obtained from TAIR^2^. We used HMMER 3.0 (Eddy, 1998) to identify *Arabidopsis*, tomato and pepper sequences that contained the complete bHLH domain (PF00010), using an E-value < 1e^–5^ threshold. Candidate sequences were verified using the SMART^4^ and NCBI databases^5^. Sequences with confirmed bHLH domains were retained for further analysis.

### Phylogenetic Analysis and Classification of the *CabHLH* Gene Family

The sequences of the CabHLH and AtbHLH proteins were extracted, and a multiple alignment of the sequences was performed using ClustalW 2.0 (Larkin et al., 2007). A phylogenetic tree was constructed in MEGA 7.0 using the neighbor joining (NJ) method (Tamura et al., 2007) with the following parameters: 1,000 bootstrap replicates, Poisson model, and pairwise deletion. *CabHLHs* were placed into subfamilies based on the classification of closely related *AtbHLHs* and the bootstrap support values at relevant nodes.

### Protein Properties, Conserved Motifs and Gene Structures

CabHLH protein sequences were uploaded to the ExPASy website^6^ to calculate their molecular weights (MW) and isoelectric points (pI). MEME tools^7^ v5.1.1 (Bailey et al., 2009) were used to identify up to ten conserved motifs in each CabHLH protein with an optimal motif width of 10–200 residues and all other parameters set to their default values. Intron locations were determined based on the GFF3 files of *Arabidopsis*, pepper and tomato sequences. Gene structures were drawn using TBtools v0.66833 (Chen et al., 2020).

### Chromosomal Mapping and Gene Duplication Analysis

The chromosomal positions of the *CabHLH* genes were obtained from the gene annotation file and visualized using MapGene2Chromosome^8^ v2. Within a genome, homologous gene pairs located within 100 kb on the same chromosome were considered to be tandem duplicates, whereas blocks of genes copied from one region to another were considered to be segmental duplications (Tang et al., 2008; Liu et al., 2011). Segmental and tandem duplicated gene pairs within the pepper genome and collinear gene pairs among the pepper, tomato and *Arabidopsis* genomes were identified using MCScanX with a match score of 50, a match size of 5, a gap score of -3, and an E-value of 1e^–05^ (Wang et al., 2012). The non-synonymous substitution rate (Ka) and synonymous substitution rate (Ks) were calculated using KaKs_Calculator 2.0 (Wang et al., 2010), and a collinearity map was drawn with Circos software (Krzywinski et al., 2009).

### Analysis of *cis*-Regulatory Elements

SeqKit v0.13.0 (Shen et al., 2016) was used to extract the promoter sequences of each *CabHLH* gene from the pepper genome file, 2000 bp upstream of the ATG start codon. Promoters were uploaded to the PlantCARE website^9^ (Lescot et al., 2002) to predict their *cis*-elements.

### Expression Analysis of the *CabHLH* Genes

RNA-Seq data were used to examine the expression of *CabHLH* genes in multiple tissues and in response to various abiotic stress treatments (Liu et al., 2017). The expression level of each gene was calculated as FPKM (fragments per kilobase of transcript per million mapped reads), transformed as log_2_ (FPKM + 1). Finally, expression heatmaps were generated in R v3.6.1.

Seeds of the pepper cultivar “6421,” which exhibits good heat, drought, and disease tolerance, were obtained from the Vegetable Institute of the Hunan Academy of Agricultural Sciences. Plants were grown using the substrate floating seedling method at 24/16°C with a 16 h light/8 h dark photoperiod. Following a previously published treatment protocol (Liu et al., 2017), 40-day-old replicate pepper seedlings were exposed to 200 mM NaCl (salt stress), 400 mM mannitol (drought), 10°C (cold stress), or 42°C (heat stress). Salt stress was imposed by adding NaCl to a final concentration of 200 mM in the nutrient solution, and drought stress was applied by adding D-mannitol to a final concentration of 400 mM. For heat and cold stress treatments, the seedlings were transferred to a growth chamber at 42 or 10°C, and the illumination, photoperiod, and relative humidity were identical to those in the control treatment. Leaf tissue of treated and control plants was sampled at four time points 1, 6, 12, and 24 h after treatment initiation. Samples of treated and control plants were harvested at 7:00, 12:00, and 18:00 h on the first day and at 6:00 h on the following day. Three seedlings were randomly selected and combined to create one biological replicate, and three biological replicates were collected for each treatment and time point. Samples were frozen in liquid nitrogen and stored at -80°C until further use.

Total RNA was extracted from frozen leaf samples using an RNA kit (TaKaRa, Dalian, China) and reverse transcribed into cDNA with a PrimeScript RT reagent kit (TaKaRa). The SYBR Premix Ex Taq kit (TaKaRa) was used to measure relative gene expression levels following the manufacturer’s instructions of the One-step Real-Time PCR System Time PCR Detection System (Applied Biosystems, Foster City, CA, United States). The cycling steps were 94°C for 30 s, 94°C for 10 s for 40 cycles, and 58°C for 30 s, followed by melting curve analysis at 65°C for 10 s for 61 cycles. The relative expression levels of selected genes were calculated using the 2^−ΔΔ^^Ct^ method (Schmittgen and Livak, 2008).

## Results

### Identification, Phylogenetic Analysis, Classification and Protein Properties of the *CabHLH* Genes

**FIGURE 1:**
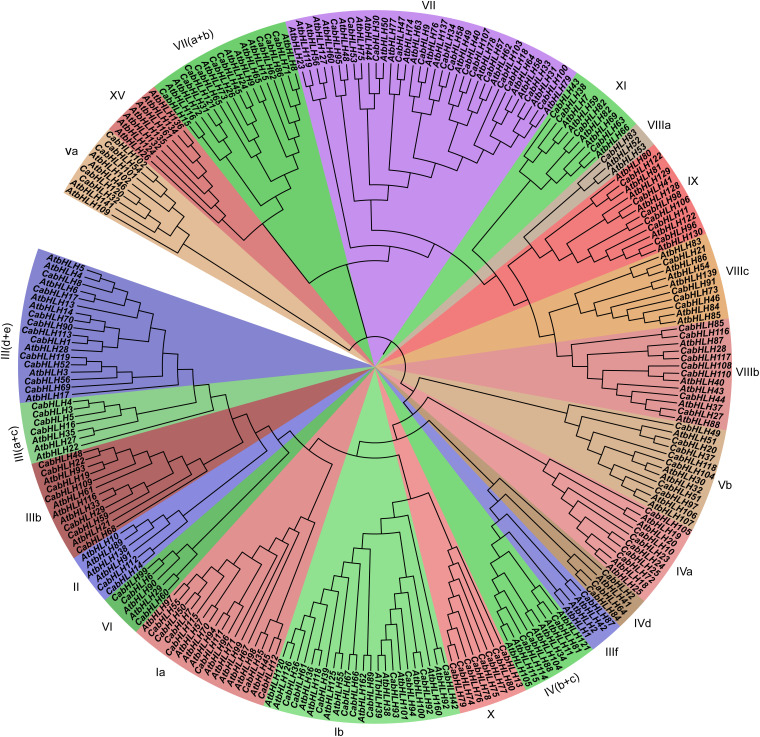
Phylogenetic analyses of bHLH proteins from pepper and *Arabidopsis*. The phylogenetic tree was constructed by MEGA7 using the neighbor-joining (NJ) method (1,000 bootstrap). Subfamilies were displayed by different colors.

We used HMMER 3.0 to search for bHLH domains (PF00010) in the pepper and tomato proteins using an E-value threshold of <1e^–5^. All candidate sequences were filtered with NCBI and SMART to further confirm that they contained complete bHLH domains. A total of 122 *CabHLH*s and 140 *SlbHLHs* were identified (Supplementary Table S1); the pepper *bHLH* genes were named *CabHLH1* to *CabHLH122* based on their arrangement on the pepper chromosomes. We constructed a phylogenetic tree of CabHLH and AtbHLH proteins in order to investigate their evolutionary relationships and to classify the *CabHLH*s into 21 established subfamilies according to the classifications of their *Arabidopsis* homologs (Li et al., 2006). Subfamily VII had the largest number of members in pepper (14 genes), whereas subfamilies IIIf and VIIIa had the fewest (one gene each) (Figure 1). Compared with *Arabidopsis*, pepper had no members of the XV subfamily but contained a unique X subfamily. In many cases, *Arabidopsis* and pepper had different numbers of genes in a given subfamily.

Comprehensive information on the *CabHLH* genes, including locus names, gene positions, protein lengths, exon numbers, molecular weights (MW), and isoelectric points (pI), is provided in Supplementary Table S2. The CabHLH proteins range in size from 117 (*CabHLH21*) to 633 (*CabHLH71*) amino acids, with an average length of approximately 344 amino acids. The MWs of the CabHLH proteins range from 12.9 kDa (*CabHLH31*) to 69.3 kDa (*CabHLH2*), and their pIs range from 4.6 (*CabHLH48*) to 10.32 (*CabHLH108*). The *CabHLH* genes contain 1 to 10 exons, highlighting the diversity of their structures.

### Chromosome Locations and Duplication Analysis of the *CabHLH* Genes

**FIGURE 2:**
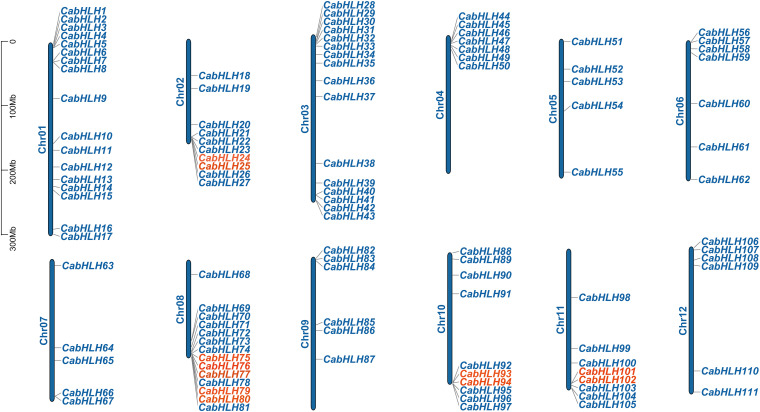
Chromosomal locations of *CabHLH* genes. Tandem duplicated genes are marked in red.

A total of 111 *CabHLH* genes were located on 12 chromosomes (91%) (Figure 2), and the other 11 genes were mapped to scaffolds (*CabHLH112*-*122*). *CabHLH* genes were unevenly distributed on the 12 chromosomes, with the largest number of *CabHLH*s on chromosome 1 (17 genes) and the smallest number on chromosomes 5 and 7 (five genes each). Chromosomes 3, 8, 2, 10, 11, 6, 4, 12, and 9 contained 16, 14, 10, 10, 8, 7, 7, 6, and 6 *CabHLH* genes, respectively. *CabHLHs* from each subfamily were also unevenly distributed among the chromosomes, and most *CabHLH*s were clustered at the ends of the chromosomes.

**FIGURE 3:**
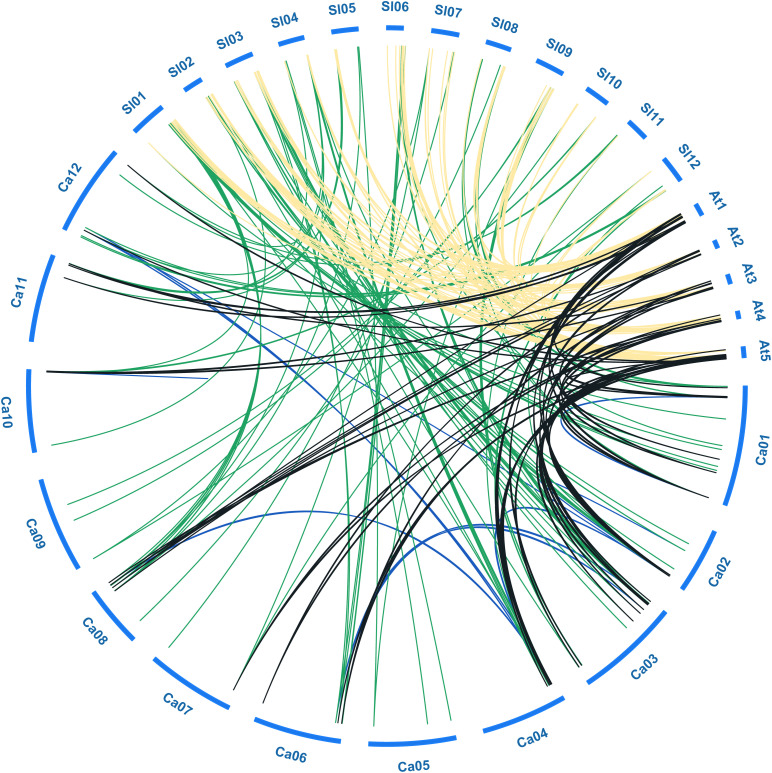
Collinear analysis of *bHLH* genes among pepper (Ca), tomato (Sl), and *Arabidopsis* (At). Green, black and yellow lines represent the collinear gene pairs between pepper and tomato, pepper and *Arabidopsis*, tomato and *Arabidopsis* chromosomes, respectively. Blue lines indicate the segmental duplicated *bHLH* genes in pepper.

We analyzed the locations of *CabHLH* duplicates in the *C. annuum* genome, as tandem duplicates and segmental duplicates play an important role in the expansion of gene families and the generation of new gene functions. As shown in Figure 2, we identified six pairs of tandem duplicates on chromosomes 2, 8, 10, and 11: *CabHLH24/25*, *CabHLH75/76*, *CabHLH76/77*, *CabHLH79/80*, *CabHLH93/94*, and *CabHLH101/102*. In addition, ten gene pairs (*CabHLH8/17*, *CabHLH19/22*, *CabHLH22/48*, *CabHLH34/58*, *CabHLH29/59*, *CabHLH46/73*, *CabHLH92/93*, *CabHLH27/108*, *CabHLH44/108*, *CabHLH48/109*) appeared to have arisen through segmental duplication (Figure 3). To determine the order of these duplication events, we used the synonymous substitution rate (Ks) to estimate the duplicate divergence times. All segmental duplicates had larger Ks values (0.83–2.32) than did tandem duplicates (0.11–0.77), indicating that they had a relatively earlier origin (Table 1). In addition, the Ks values of *CabHLH46/73* and *CabHLH27/108* were significantly greater than those of other segmental duplicates, showing that they derived from more ancient duplication events.

**TABLE 1:** Calculation of Ka and Ks ratios of 16 duplicated *CabHLH* gene pairs.

Genes	Ka	Ks	Duplication Type
CabHLH24/CabHLH25	0.2381	0.7698	Tandem
CabHLH75/CabHLH76	0.1017	0.1502	Tandem
CabHLH76/CabHLH77	0.0814	0.1058	Tandem
CabHLH79/CabHLH80	0.1069	0.2141	Tandem
CabHLH93/CabHLH94	0.1550	0.2800	Tandem
CabHLH101/CabHLH102	0.1081	0.1526	Tandem
CabHLH8/CabHLH17	0.1546	1.0551	Segmental
CabHLH19/CabHLH22	0.2626	1.0137	Segmental
CabHLH22/CabHLH48	0.4339	1.3220	Segmental
CabHLH34/CabHLH58	0.2341	0.8915	Segmental
CabHLH29/CabHLH59	0.2142	0.9823	Segmental
CabHLH46/CabHLH73	0.4674	2.3151	Segmental
CabHLH92/CabHLH93	0.4884	0.9128	Segmental
CabHLH27/CabHLH108	0.4385	2.1414	Segmental
CabHLH44/CabHLH108	0.3526	1.1955	Segmental
CabHLH48/CabHLH109	0.2438	0.8300	Segmental

To further explore the evolutionary relationships among bHLH TFs from different species, we constructed a collinearity plot of the pepper, tomato, and *Arabidopsis* bHLH gene families (Figure 3). A total of 117, 64, and 105 collinear gene pairs were identified between pepper and tomato, pepper and *Arabidopsis*, and tomato and *Arabidopsis*, respectively, indicating that significant expansion of the gene family had occurred before divergence of the three species (Supplementary Table S3). For example, 44 *CabHLH*s and 54 *AtbHLH*s had a collinear relationship, and most such relationships were one-to-one matches such as *CabHLH2/AtbHLH2* and *CabHLH12/AtbHLH45*. There were also one-to-many matches, such as *CabHLH17*/(*AtbHLH4, AtbHLH5, AtbHLH6*) and *CabHLH23*/(*AtbHLH18, AtbHLH25*). Many-to-one cases also existed, such as (*CabHLH6, CabHLH8, CabHLH17*)/*AtbHLH4* and (*CabHLH27, CaHLH108, CabHLH44*)/*AtbHLH88*. These results indicate that *bHLH*s are relatively conserved and that collinear *bHLH*s between species may originate from the same ancestor.

### Gene Structure and Motif Analysis of CabHLH Family

**FIGURE 4:**
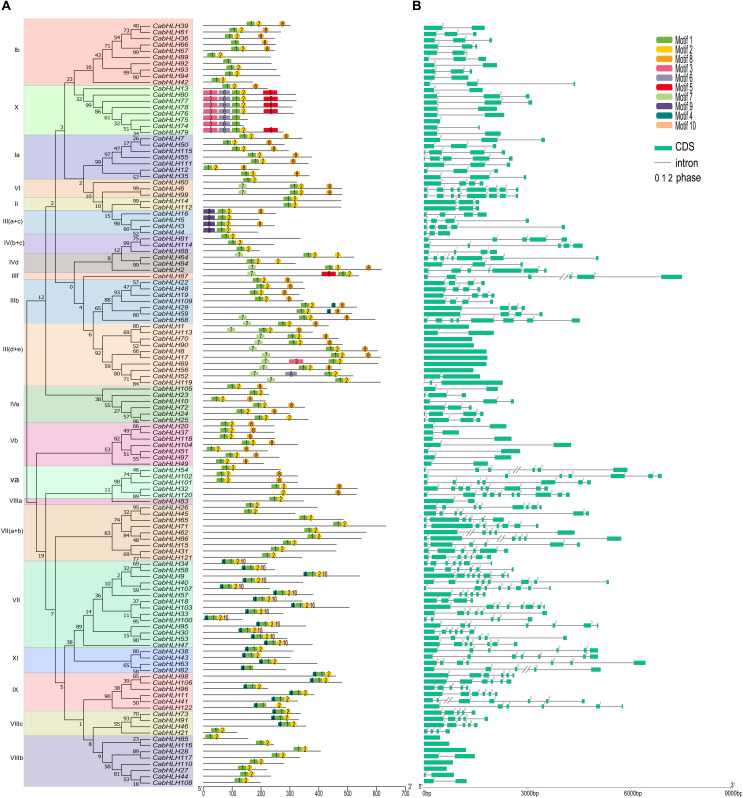
Exon–intron structures of *CabHLH* genes and conserved motifs of CabHLH proteins. **(A)** Exon–intron organization of *CabHLH* genes. Green boxes represent exons and black lines indicate introns. The numbers 0, 1, and 2 denote the intron phases. **(B)** Conserved motifs in the CabHLH proteins. The conserved motifs were identified using MEME with complete protein sequences. Different motifs are displayed by various colors.

Conserved motifs of the CabHLH proteins were analyzed using MEME tools, and ten conserved motifs from 26 to 154 amino acids in length were identified (Supplementary Figure S1). The number of conserved motifs in each CabHLH protein varied from one to five (Figure 4). Each subfamily contained several common motifs, while few subfamilies possessed unique motifs. For example, motifs 1 and 2 were present in almost all CabHLH proteins and represented the position of the bHLH domain, whereas motifs 9 and 10 were only found in subfamilies III (a + c) and VII, respectively, and may be related to unique functions of individual subfamilies. CabHLH proteins from the same subfamily exhibited similar motifs, suggesting that they may also share a degree of functional similarity. The diversity of motifs in different subfamilies suggests that *CabHLH* functions have tended to diversify during evolution.

We used TBtools to map the structures of the pepper, tomato and *Arabidopsis bHLH* genes (Figure 4, Supplementary Figure S2) and found that most *bHLHs* from the same subfamily shared similar gene structures. For example, subfamily III (d + e) contains 0–2 introns, subfamily IX has 4–6 introns. Intron gain and loss is a frequent phenomenon during evolution and can increase the complexity of gene structures (Roy and Gilbert, 2005). In the *CabHLHs*, most tandem duplicates (5/6) had different numbers of introns, whereas most segmental duplicates (7/10) had the same number of introns, suggesting that tandem duplicates may have undergone greater divergence in gene function over the course of evolution. In addition, we also analyzed the introns of collinear *bHLH* pairs. There were 53, 31, and 46 pairs of collinear *bHLH* pairs with different numbers of introns between pepper and tomato, pepper and *Arabidopsis*, and tomato and *Arabidopsis*, respectively, indicating that the functions of these collinear genes may have undergone a degree of differentiation (Supplementary Table S3).

### *Cis*-Element Analysis of the *CabHLH* Genes

We extracted the 2,000 bp upstream promoter sequences of the *CabHLH* genes for *cis*-element analysis using the PlantCARE database. Ten common *cis*-elements were identified (Supplementary Table S4), and 119 *CabHLHs* contained at least one *cis*-element. The ABRE, CGTCA-motif, and GARE-motif elements respond to ABA, JA, and GA stimulation, These motifs were present in the promoters of 86, 77, and 27 *CabHLHs*, suggesting that the expression of these genes responds to levels of the corresponding hormones. Two light-responsive elements (G-box and Sp1) that are ubiquitous in plants were identified in 90 and 12 *CabHLHs*, respectively. Stress-responsive cis-elements, including those associated with LTR, defense and stress (TC-rich repeats), drought (MBS), and anaerobic induction (ARE), were identified in the promoters of 33, 42, 52, and 27 *CabHLHs*, respectively. Diverse response elements indicate the importance of *CabHLHs* in stress responses.

### Expression Patterns of *CabHLH* Genes in Various Tissues

**FIGURE 5:**
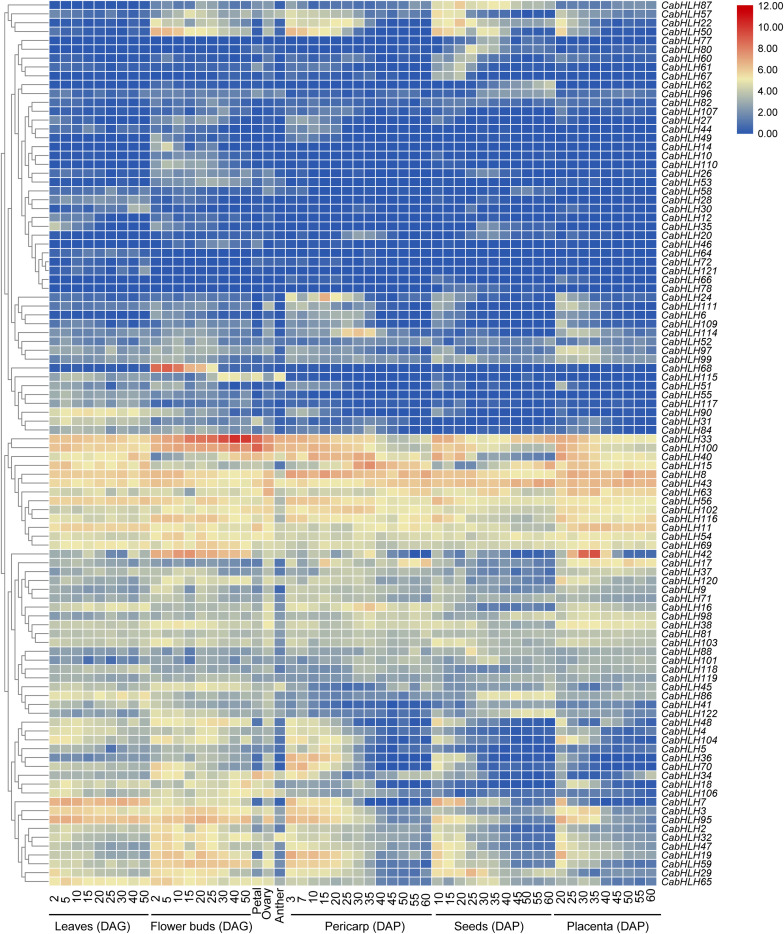
Expression patterns of *CabHLH* genes in different tissues and organs. Heatmap of expression profiles [in log_2_ (FPKM + 1)] for *CabHLH* genes in different tissues and organs. The expression levels are displayed by the color bar. DAG, days after germination; DAP, days after pollination.

**FIGURE 6:**
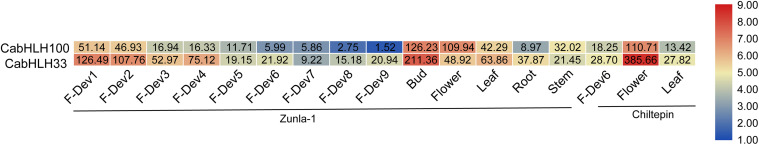
Heatmap of expression profiles [in log 2 (RPKM + 1)] of *CabHLH33* and *CabHLH100* in two pepper cultivars “Zunla-1” (*Capsicum annuum* L.) and “Chiltepin” (*C. annuum var. glabriusculum*). The expression levels are displayed by the color bar. F-Dev-1, F-Dev-2, F-Dev-3, F-Dev-4, and F-Dev-5 (0–1 cm, 1–3 cm, 3–4 cm, 4–5 cm, and mature green fruit), F-Dev-6 (fruit turning red), F-Dev-7, F-Dev-8, and F-Dev-9 (3, 5, and 7 days after turning red). RPKM, reads per kilobase million.

We obtained the expression data of *CabHLH* genes from previous research (Liu et al., 2017) and removed 22 *CabHLH* genes with FPKM values of less than one in all tissues (Zhuo et al., 2018). An expression heatmap was created using the remaining 100 genes (Figure 5, Supplementary Table S5). Most *CabHLH*s differed in their expression patterns, although a few showed similar expression patterns. Some *CabHLH*s (such as *CabHLH100, CabHLH11, CabHLH8*, and *CabHLH43*) showed high expression levels (FPKM > 10) in most tissues analyzed, whereas other *CabHLH*s (such as *CabHLH23, CabHLH85, CabHLH39, CabHLH105*, and *CabHLH108*) were not expressed in any tissues. In addition, several *CabHLHs* showed extremely high expression in specific tissues, such as *CabHLH33*/*CabHLH100* in flower buds, *CabHLH33* in petals, and *CabHLH42* in the placenta. We obtained transcriptome data for *CabHLH33* and *CabHLH100* from another study and found that their expression was also significantly higher in flowers or flower buds than in any other tissues (Figure 6) (Qin et al., 2014). These genes may therefore have specific roles in flower development. We also analyzed the expression of duplicated genes in various tissues and found that most duplicated gene pairs had similar expression patterns, such as *CabHLH48*/*CabHLH109*, *CabHLH75/CabHLH76*, and *CabHLH46/CabHLH73*, which were expressed at low levels in most tissues. By contrast, the expression of *CabHLH34* in flowers was much higher than that of its duplicate *CabHLH58*, and the expression of *CabHLH8* was higher than that of its duplicate *CabHLH17* in all tissues analyzed.

### Expression Analysis of *CabHLH* Genes Under Abiotic Stresses

**FIGURE 7:**
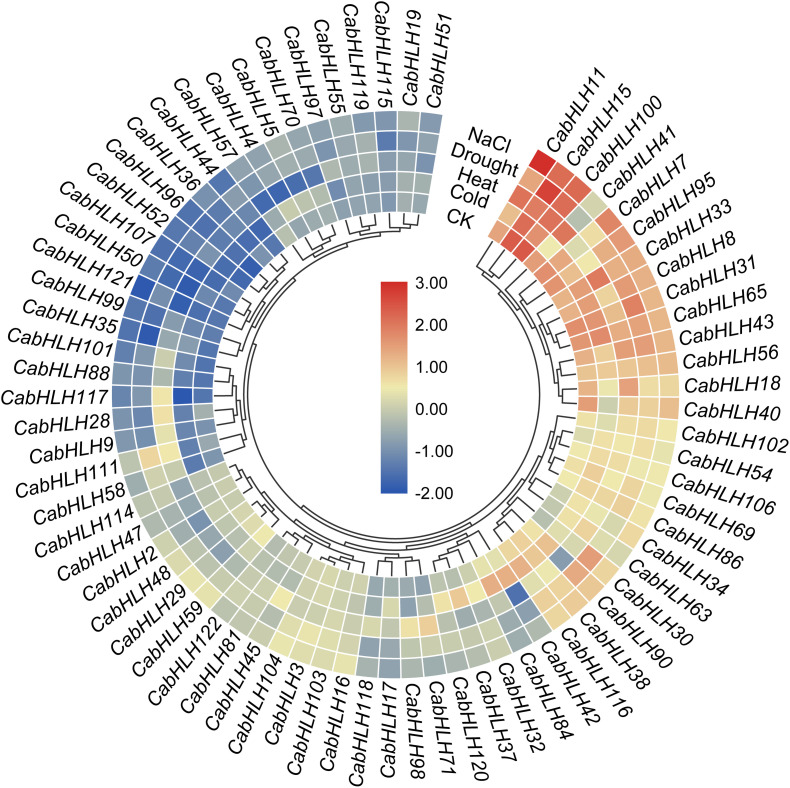
Expression heatmap of *CabHLH* genes under multiple abiotic treatments. The color scale represents log_2_ (FPKM + 1) values.

To analyze the response of the *CabHLH* genes to abiotic stress, we extracted transcriptome data for *CabHLH* gene expression after 6 h of cold, heat, salt, and drought stress. We used genes with FPKM values of bigger than one in at least one group to create a clustered heatmap and found many *CabHLHs* responded to abiotic stress (Figure 7, Supplementary Table S6). We also analyzed the relationship between transcriptome data and *cis*-elements and found that gene expression results were not clearly correlated with the presence/absence of specific *cis*-elements. For example, some *CabHLHs* with LTR promoter elements, such as *CabHLH5/17/32/65/90*, were upregulated under LTR treatment. However, some *CabHLHs* with LTR elements, such as *CabHLH16/36/48/114*, were downregulated or remained unchanged (Figure 7). This result indicates that the expression of these *CabHLHs* might induced by several *cis*-elements, and unidentified *cis*-elements might contribute to regulating the expression of these *CabHLHs* under abiotic stress.

**FIGURE 8:**
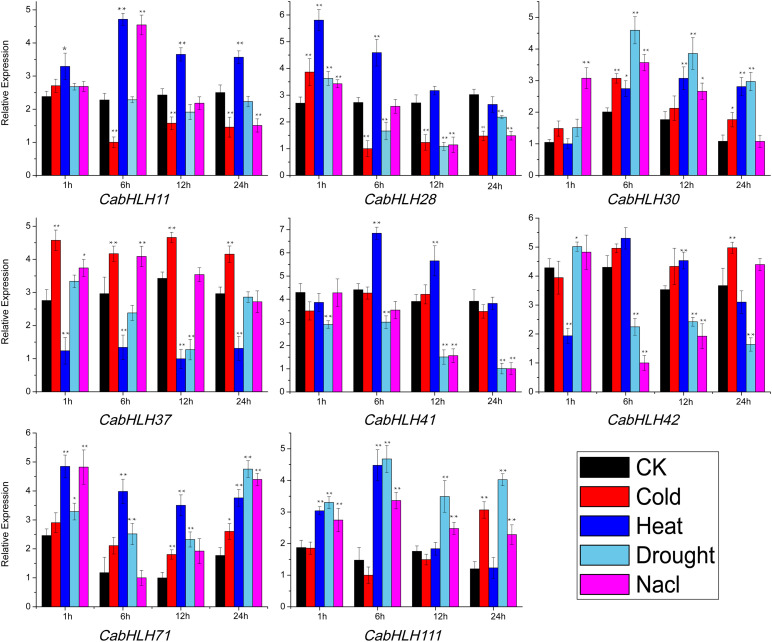
qRT-PCR analysis of *CabHLH* genes under cold, heat, salt and drought treatments following a 24 h time course. *y*-axis: relative expression levels; *x*-axis: the time (hours) course of stress treatments. *t*-test: one asterisk denotes significant differences (*P* < 0.05) between treatment group and control group (CK); two asterisks denote extremely significant differences (*P* < 0.01).

To further validate the effects of abiotic stress on the expression of *CabHLH* genes, we selected eight genes that responded to abiotic stress (Supplementary Table S6) and verified the expression patterns of these genes using qRT-PCR (Figure 8). The specific primers used are listed in Supplementary Table S7. After cold stress treatment, the expression levels of *CabHLH30*, *CabHLH37*, *CabHLH42*, *CabHLH71*, and *CabHLH111* were upregulated, *CabHLH11* was downregulated, *CabHLH28* was first upregulated and then downregulated, and *CabHLH41* remained unchanged. After high temperature treatment, the expression levels of *CabHLH37* and *CabHLH42* were downregulated, and the expression levels of the remaining genes were upregulated. After drought treatment, *CabHLH30*, *CabHLH71*, and *CabHLH111* were upregulated, *CabHLH41* and *CabHLH37* were downregulated, and *CabHLH42* was first upregulated and then downregulated. Under salt stress, the expression levels of *CabHLH30*, *CabHLH37*, *CabHLH71*, and *CabHLH111* were upregulated, *CabHLH11* and *CabHLH28* were first upregulated and then downregulated, *CabHLH41* and *CabHLH42* were downregulated. In general, there was good correspondence between the RNA-seq data and the qRT-PCR results. However, few exceptions existed. For example, in the qRT-PCR experiment, the expression level of *CabHLH11* decreased after 6 h of cold treatment, but its expression was unchanged in the RNA-seq analysis, perhaps due to different sampling time points (qRT-PCR at 12:00, RNA-seq at 14:00).

## Discussion

A growing body of evidence suggests that plant *bHLH* genes are involved in physiological and biochemical processes such as stress resistance, growth and development, biosynthesis, and signaling (Duek and Fankhauser, 2003; Hernandez et al., 2004; Castillon et al., 2007). Members of the bHLH TF family have been identified in *Arabidopsis* (Toledo-Ortiz et al., 2003), rice (Li et al., 2006), apple (Yang et al., 2017), cabbage (Song et al., 2014), tomato (Sun et al., 2015), ginseng (Chu et al., 2018), and other species by comparative genomics. Until now, this family had not been characterized in pepper. In this study, we systematically analyzed the pepper bHLH TF family and provided a reference for further exploration of the roles of bHLH genes in regulation of pepper growth and stress responses.

A total of 122 *CabHLH* genes were identified and classified into 21 subfamilies according to their phylogenetic relationships with known *bHLH* genes from *Arabidopsis* (Li et al., 2006). Compared with *Arabidopsis*, pepper lacks members of the XV subfamily but contains a unique X subfamily. The acquired genes may counter gene losses, or even evolve novel functions (Qian et al., 2010). The functions of some *AtbHLH*s have been identified in previous studies. For example, *AtbHLH15* and *AtbHLH8* from subfamily VII (a + b) can combine with active phytochromes and mediate light signaling responses (Castillon et al., 2007). *AtbHLH44*, *AtbHLH58*, and *AtbHLH50* in subfamily VII are early response BR signaling components required for full BR response (Friedrichsen et al., 2002). Overexpression of *AtbHLH116* from subfamily IIIb in wild-type plants improves the expression of the *CBF* regulon in the cold and enhances freezing tolerance of transgenic plants (Chinnusamy et al., 2003). *AtbHLH1* from subfamily IIIf encodes a bHLH protein that regulates trichome development in *Arabidopsis* through interaction with GLABRA3 and TESTA GLABRA1 (Payne et al., 2000). *CabHLH*s and *AtbHLH*s from the same subfamilies may have similar functions, although this will require further experimental verification.

Gene duplication, including tandem duplication and segmental duplication, is the most important pathway for the evolution and expansion of gene families (Vision et al., 2000). We identified six tandem duplicated *CabHLH*s and ten segmental duplicated *CabHLH*s in the pepper genome. Collinear genes derive from a common ancestor and are present in the same relative positions in the genomes of two or more species. We identified 117, 64 and 105 collinear *bHLH* pairs between pepper and tomato, *Arabidopsis* and pepper, and *Arabidopsis* and tomato, respectively. In the process of evolution, collinear blocks may be disrupted by various factors. The greater the evolutionary distance, the fewer collinear gene pairs will be identified between species, and collinearity can therefore be used as a measure of the evolutionary distance between species (Wicker et al., 2010). There were more collinear gene pairs between tomato and pepper, consistent with the fact that both are members of the Solanaceae family (Qin et al., 2014). Previous studies have shown that the amplification of transposable elements has eroded collinearity in the pepper genome (Wicker et al., 2010; Qin et al., 2014), which may explain why the number of collinear gene pairs between pepper and *Arabidopsis* is much lower than that between *Arabidopsis* and tomato.

We identified ten highly conserved motifs in the CabHLH proteins. Similar to the bHLHs of potato, lotus and *Arabidopsis* (Wang et al., 2018; Mao et al., 2019), motif 1 and motif 2 were present in almost all CabHLH proteins and represented the position of the bHLH domain, which is highly conserved among species. However, motif 9 and 10 were only present in subfamilies III (a + c) and VII, respectively. Variation in conserved motifs permits the classification of proteins into subfamilies and reflects each subfamily’s specific functions (Jiang et al., 2019). Gene structure can also provide information for the study of gene family evolution (Guo et al., 2013). The number of introns varied from 0 to 9, indicating that the gain and loss of introns had occurred, which may be another reason for the differences among CabHLH subfamilies (Paquette et al., 2000).

We analyzed the expression profiles of *CabHLH*s in different tissues and found a large variety of expression patterns. Some *CabHLHs* (such as *CabHLH100, CabHLH11, CabHLH8*, and *CabHLH43*) were highly expressed (FPKM > 10) in most tissues analyzed and may participating in various development processes of pepper. Several *CabHLHs* were highly expressed in specific tissues, suggesting that they may have a role in those tissues’ development. For example, *CabHLH33*, a homolog of *AtbHLH31*, was highly expressed in flower buds and petals. Previous studies suggest that *AtbHLH31* regulates petal growth by controlling cell expansion (Varaud et al., 2011), and *CabHLH33* may have a similar function in pepper. However, *CabHLH*s that were not expressed in any tissues (such as *CabHLH23*, *CabHLH85*, *CabHLH39*, *CabHLH105*, and *CabHLH108*) may have lost their functions during evolution and become pseudogenes, as has been demonstrated in the evolution of other plant genomes (Innan and Kondrashov, 2010; Xie et al., 2019). In addition, several duplicated pairs (such as *CabHLH34/CabHLH58* and *CabHLH8/CabHLH17*) had significantly different expression patterns, indicating that functional diversification of duplicated *CabHLH* pairs had occurred during the course of evolution (Blanc and Wolfe, 2004).

Plant *bHLH*s modulate stress responses, including responses to LTR (Feng et al., 2012; Lin et al., 2014; Xu et al., 2014), drought (Li et al., 2007; Seo et al., 2011), heat (Ko et al., 2009), and salt (Murre et al., 1989; Zhou et al., 2009; Liu et al., 2014). In this work, *cis*-element analysis indicated that *CabHLH*s contained elements (such as LTR, ABRE, and TC-rich) that are responsive to various stresses, which was consistent with previous research on potato and lotus *bHLH*s (Wang et al., 2018; Mao et al., 2019). In addition, we extracted the transcriptome data of *CabHLH*s and performed a qRT-PCR experiment to validate the response of the *CabHLH* genes to abiotic stress. These upregulated *CabHLH*s (such as *CabHLH30/37/42/71/111* under cold, *CabHLH11/28/30/41/71/111* under heat, *CabHLH30/71/111* under drought, and *CabHLH30/37/71/111* under NaCl) may regulate downstream bHLH-related genes, thus enhancing the stress tolerance of pepper. Our research provides a framework for further functional characterization of *CabHLH* genes.

## Data Availability Statement

All datasets presented in this study are included in the article/Supplementary Material.

## Author Contributions

ZZ, FL, XH, and XZ conceived and designed the experiments. ZZ, JC, and CL performed the experiments. ZZ, CL, FL, and XH analyzed data. ZZ and XZ wrote the manuscript. All authors read and approved the manuscript.

## Conflict of Interest

The authors declare that the research was conducted in the absence of any commercial or financial relationships that could be construed as a potential conflict of interest.

## Footnotes

1 https://solgenomics.net/

2 https://www.Arabidopsis.org/

4 ^3^ http://smart.embl-heidelberg.de/

5 http://www.ncbi.nlm.nih.gov/

6 http://web.expasy.org/

7 http://meme-suite.org

8 http://mg2c.iask.in/mg2c_v2.0/

9 http://bioinformatics.psb.ugent.be/webtools/plantcare/html/

## Supplementary Material

FIGURE S1 Sequence logos of conserved motifs of CabHLHs.

FIGURE S2 Exon-intron structures of *AtbHLHs* and *SlbHLHs*. The phylogenetic tree of *AtbHLHs* and *SlbHLH* proteins was constructed by MEGA7 using the neighbor-joining (NJ) method (1,000 bootstrap). Green boxes represent exons and black lines indicate introns.

TABLE S1 Genes with complete bHLH domain in pepper, tomato, and *Arabidopsis*.

TABLE S2 Basic information of the CabHLH transcription factor family.

TABLE S3 Collinear *bHLHs* among tomato, pepper and *Arabidopsis*.

TABLE S4 *Cis*-elements analysis of the *CabHLH* genes.

TABLE S5 The FPKM values of *CabHLH* genes in different tissues and organs.

TABLE S6 FPKM values of *CabHLH* genes under salt, drought, cold, and heat stress.

TABLE S7 Primer sequences used in qRT-PCR.
