# Supplementary figures and images for: Genome-Wide Identification and Characterization of the bHLH Transcription Factor Family in Pepper (Capsicum annuum L.)
Source: Front Genet. 2020 Sep 25;11:570156. doi: 10.3389/fgene.2020.570156 (PMC7545091; doi:10.3389/fgene.2020.570156)

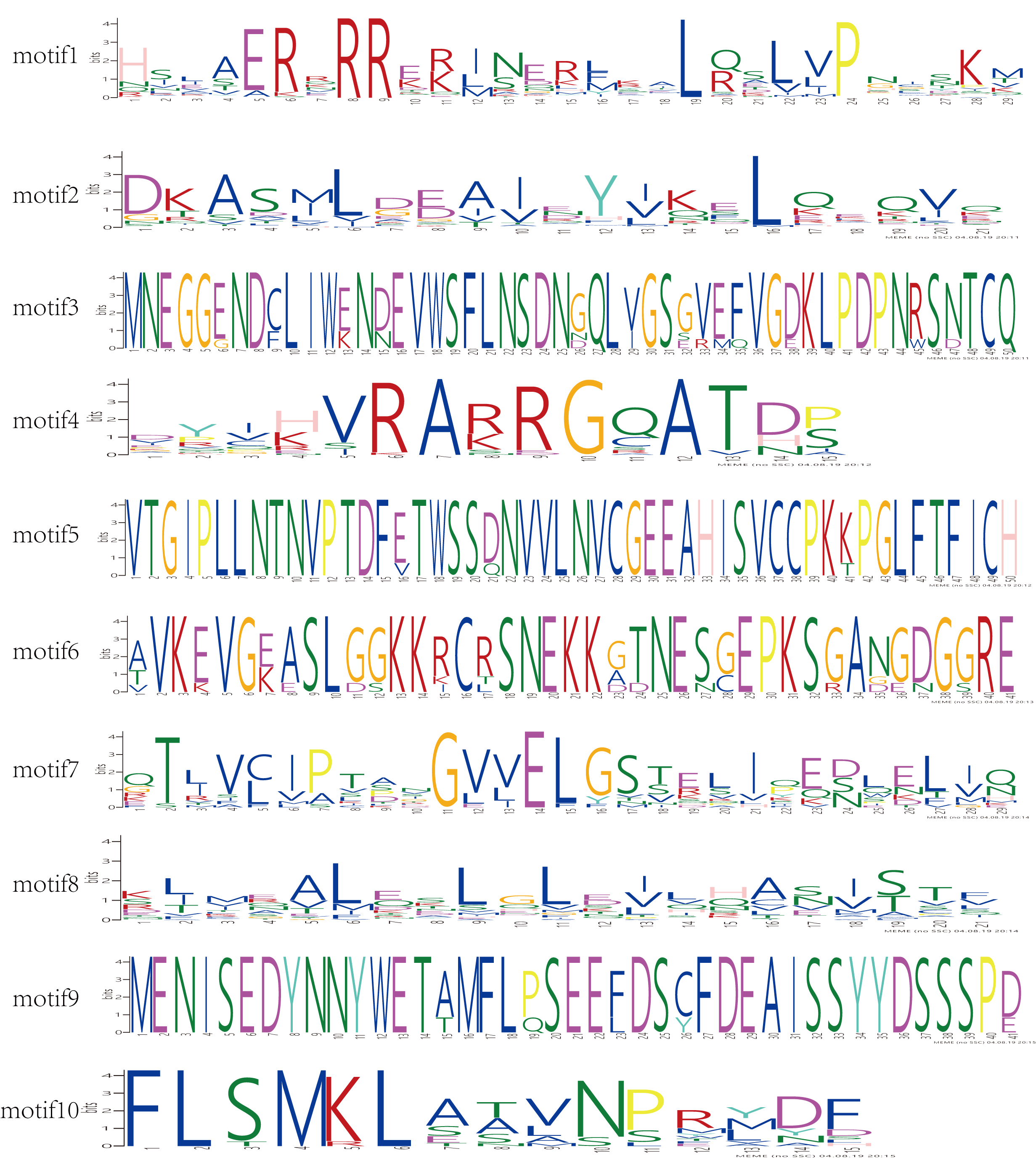

Supplement: FIGURE S1 — Sequence logos of conserved motifs of CabHLHs. [file Image_1.TIF]

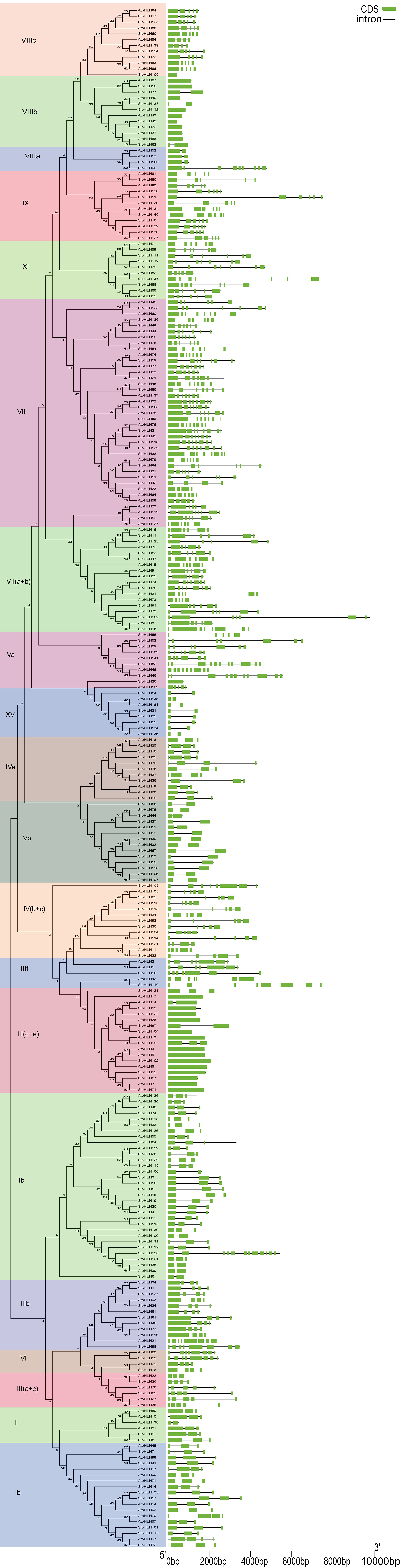

Supplement: FIGURE S2 — Exon-intron structures of AtbHLHs and SlbHLHs. The phylogenetic tree of AtbHLHs and SlbHLH proteins was constructed by MEGA7 using the neighbor-joining (NJ) method (1,000 bootstrap). Green boxes represent exons and black lines indicate introns. [file Image_2.TIF]
